# Community Psychiatry and Ageing: A Narrative Review on Addressing Depression, Cognitive Impairment and Social Isolation through Community-Based Policies

**DOI:** 10.1192/j.eurpsy.2026.10780

**Published:** 2026-07-31

**Authors:** Â. Ferreira, J. Marta, C. Batista, J. Miranda, M. M. Figueiredo, C. Ramos, D. Durães

**Affiliations:** Arrábida’s Local Health Unit, Setúbal, Portugal

## Abstract

**Introduction:**

The global population is ageing rapidly, with the proportion of adults over 65 projected to double by 2050. Mental health in older adults is often under-recognised, despite high prevalence of depression (≈7%) and dementia (≈5%) worldwide. Social isolation and loneliness are major determinants of late-life psychiatric morbidity, increasing risk for depression, cognitive decline and premature mortality. Traditional models of mental health care for older adults remain hospital-centred and fragmented, limiting access and continuity.

**Objectives:**

To review the role of community psychiatry and community-based policies in promoting mental health among older adults, focusing on interventions addressing depression, cognitive impairment and social isolation.

**Methods:**

Narrative synthesis of epidemiological studies, community interventions and policy frameworks (2010–2024). Emphasis was placed on programmes integrating psychiatry, primary care, social services and local community resources.

**Results:**

Community-based mental health programmes targeting older adults show positive outcomes. Collaborative care models integrating psychiatry and primary care significantly improve depression treatment response in late life (OR≈1.5). Social prescribing initiatives, where general practitioners link isolated elders to community activities, reduce loneliness and improve quality of life. Age-friendly community policies, promoted by WHO, demonstrate reduced cognitive decline when combined with early detection and memory clinics embedded in primary care. Digital and peer-support interventions also show feasibility, particularly when adapted for accessibility and cultural context. Importantly, success depends on cross-sector collaboration between mental health services, municipalities, NGOs and older adults themselves.

**Conclusions:**

Community psychiatry is essential to meet the mental health needs of ageing societies. By embedding services within primary care, leveraging social prescribing and promoting age-friendly policies, community psychiatry addresses depression, cognitive decline and social isolation in older adults. This “whole society” approach ensures inclusivity, reduces fragmentation and strengthens resilience in ageing populations.

**Disclosure of Interest:**

None Declared

